# Groundcover improves nutrition and growth of citrus trees and reduces water runoff, soil erosion and nutrient loss on sloping farmland

**DOI:** 10.3389/fpls.2024.1489693

**Published:** 2024-11-06

**Authors:** Rui Liu, Yuting Zhang, Zhichao Wang, Xueliang Zhang, Wenjing Xu, Jianwei Zhang, Yueqiang Zhang, Bin Hu, Xiaojun Shi, Heinz Rennenberg

**Affiliations:** ^1^ Center of Molecular Ecophysiology (CMEP), College of Resources and Environment, Southwest University, Chongqing, China; ^2^ College of Resources and Environment, Southwest University, Chongqing, China; ^3^ Interdisciplinary Research Center for Agriculture Green Development in Yangtze River Basin, Southwest University, Chongqing, China

**Keywords:** citrus orchard, leaf nutrients, tree growth, groundcover management, purple soil, runoff, soil and nutrient losses

## Abstract

**Introduction:**

Groundcover management plays a crucial role in improving water retention and soil nutrition in orchard systems, thereby preventing environmental constrains by non-point source pollution. However, effectiveness of groundcover management in citrus orchards developed on sloping farmland with eroded purple soil has not been studied in detail. In particular, information on the soil nutrient losses, *e.g.*, nitrogen (N) and phosphorus (P), through interflow and its effects on growth and nutrition of citrus plants has not been reported.

**Methods:**

The present study evaluated the effects of different cover crops, *i.e.*, *Lolium perenne* L. (Lolium), *Vicia villosa* Roth (Vicia) and *Orychophragmus violaceus* (Ory), on nutrition and growth of citrus trees as well as water, soil and nutrient retention in an orchard developed in sloping farmland during two consecutive years.

**Results and discussion:**

The results show that the groundcover species Lolium and Vicia mediated nursing effects on nutrition and growth of citrus trees. These nursing effects included enhanced foliar levels of carbon(C), N and P as well as increased tree height, stem diameter, and crown width. Groundcover management generally reduced the annual surface runoff, interflow, soil loss, total N loss and total P loss. Among the cover crop species studied, Lolium and Vicia were overall more efficient than Ory in this context. Lolium reduced the average annual total loss of N and P by 42.53% and 49.23%, respectively, compared with clean tillage. The estimated annual reduction potentials of soil, N and P losses in Southwestern China were 16.3, 3.4 and 8.5 million tons yr^-1^, respectively. Obviously, Lolium and Vicia provide highly beneficial ground coverage on sloping farmland and, thus, can be used for future sustainable development of citrus orchards.

## 1 Introduction

Land degradation by erosion is a critical environmental problem worldwide that threatens global soil fertility and sustainable agriculture (Prosdocimi et al., 2016; Xiong et al., 2018; Poesen, 2018). This issue is especially significance in Southwest China, where poor agricultural practices under extreme rainfall have caused widespread soil erosion and nutrient leaching (Su et al., 2022). Nitrogen (N) and phosphorus (P) losses via runoff are major contributors to agricultural non-point source pollution, environment, and human health (Bowles et al., 2018; Liu et al., 2021). Particularly, orchards grown on farmland slopes are among the most severe areas to these losses (Wang et al., 2018; Du et al., 2021).

Farmland slopes on purple soil in Southwest China have been facing severe soil, water, and nutrient losses due to hilly terrain, high precipitation, and unsustainable cultivation practices. These farmland slopes cover 7.67 million ha, accounting for ca. 32.0% of sloping land nationwide (Zhang et al., 2016; Dai et al., 2018). Effective land management, such as groundcover, is essential to control runoff, reduce erosion, and improve nutrient status on degraded and/or marginal farmland (Zhang et al., 2016). Moreover, interflow is a major pathway for N and P losses from these systems, making it crucial to manage nutrient leaching and promote sustainable orchard management.

Citrus orchards on purple soil farmland slopes are economically important in Southwest China. However, the space between rows of citrus trees was usually bare due to herbicide application as a common farming practice, thus lacking surface vegetation cover. This is of particular significance for these farming systems, because purple soil in this area is characterized by steep slopes, a shallow soil layer, high gravel content, weak water and fertilizer retention (Wang et al., 2020a; Liang et al., 2021). The dual structure of rock and soil aggravates the risk of soil, water and fertilizer loss and intense nutrient leaching in orchards on these farmland slopes (Zhang et al., 2016; Zhong et al., 2019; Wang et al., 2021). Groundcover is a promising strategy to prevent soil, water, and fertilizer losses in orchard ecosystems worldwide. Compared with conventional clean tillage, groundcover has numerous advantages for nutrient cycling and sustainable development of orchard ecosystems. These advantages include: improved soil physical and chemical properties and nutrient preservation (Liu et al., 2021; de Torres et al., 2021); increased soil organic carbon content and fertility (Zheng et al., 2021; Reis et al., 2024); increased soil microbial diversity to promote soil nutrient cycling (Wang et al., 2020b; Wu et al., 2021); and improved yield and quality of fruit tree products (Tu et al., 2021; Panigrahi et al., 2021). Additionally, groundcover has been shown to significantly reduce soil erosion and nutrient losses in orchards compared to conventional clean tillage (Martinez-Mena et al., 2020; Garcia et al., 2018).

Although in previous studies, efficiency of groundcover management in orchard ecosystems in controlling soil, water and nutrient losses were reported, such results were either not generated in studies on purple soil, and/or not on the degraded sloping farmland systems. For instance, on yellow cinnamon soil slopes of reservoir area of central China, runoff was reduced by 31% and soil loss was reduced by 20% through groundcover (Liu et al., 2012). In an avocado orchard on Calcisol soil of Southeast Spain, groundcover reduced soil loss and runoff by 56% and 62%, respectively (Almagro et al., 2016). Research on red soil citrus orchards of Southeast China indicated that groundcover reduced runoff and soil loss by 89% and 99%, respectively (Mo et al., 2019). Thus, published information indicates that the effect of groundcover on runoff and nutrient losses differs significantly between soil types. In addition, previous studies on the characteristics of nutrient output from sloping land mainly focused on surface flow, but information on nutrient loss through interflow is lacking (Zheng et al., 2017). Additionally, while interflow is predicted to be a significant source of N losses in purple soil, a comprehensive investigation comparing nutrient losses via surface runoff and interflow has yet to be conducted (Han et al., 2010; Song et al., 2017).

The effects of groundcover on orchard tree growth and nutrition are economically and ecologically important but underexplored, especially in citrus orchards on purple soil. For instance, milk vetch cultivation in Southern China paddy fields significantly improved soil quality and rice yield (Zhou et al., 2020), while groundcover on North China’s brown soils enhanced soil conditions and the growth of *Juglans regia* (walnut) trees (Dong et al., 2021). However, cover crops can also compete with trees for nutrients and water, as observed in a *Abies fraseri* (Fraser fir) plantation in Michigan, USA, where groundcover reduced leaf N and P content in trees (Nikiema et al., 2012). The effects of groundcover on growth and nutrition in citrus orchards on purple soil of degraded sloping farmland have not been reported. In this context, legumes are particularly interesting as groundcover species. Since symbiosis of legume roots with rhizobia mediates the use of atmospheric N_2_ by biological nitrogen fixation (BNF) as N source for plant growth and development (Frungillo, 2022), groundcover by legumes may constitute a cost-effective and attractive alternative to fertilization for the improvement of N and P availability in the groundcover-orchard tree-soil system (Hao et al., 2014). Such nursery effects of legumes are of particular significance for the successful establishment and growth of non-N_2_-fixing tree species in mixed cultivation on N depleted soil (Hu et al., 2017).

In the present study, we conducted a 2-years consecutive field experiment aimed at comparing the effects of different groundcover management (*i.e.*, clean tillage, coverage with *Lolium perenne* L., *Vicia villosa* Roth, or *Orychophragmus violaceus*) on citrus tree nutrition and growth as well as surface runoff and interflow with particular emphasis on soil erosion, water retention as well as N and P losses in citrus orchards on sloping farmland of the purple soil regions in Southwest China. The objectives of this study were to (i) elucidate the effects of different groundcover species on nutrition and growth of citrus trees; (ii) quantify the efficiency of groundcover species in reducing soil erosion, water runoff, N and P losses; and (iii) select a ground cover crop, suitable for maintaining growth and nutrition of citrus trees and maximizing the reduction of runoff, soil and nutrient losses. Based on these objectives, we hypothesized that (i) groundcover improves nutrition and growth of citrus trees; and (ii) reduces soil, water, N and P losses, and the need for fertilizer application; (iii) the losses of N and P in interflow are much higher than those in surface runoff; and (iv) these effects of groundcover management highly depend on the cover crop species selected.

## 2 Materials and methods

### 2.1 Background of study area

**Figure 1:**
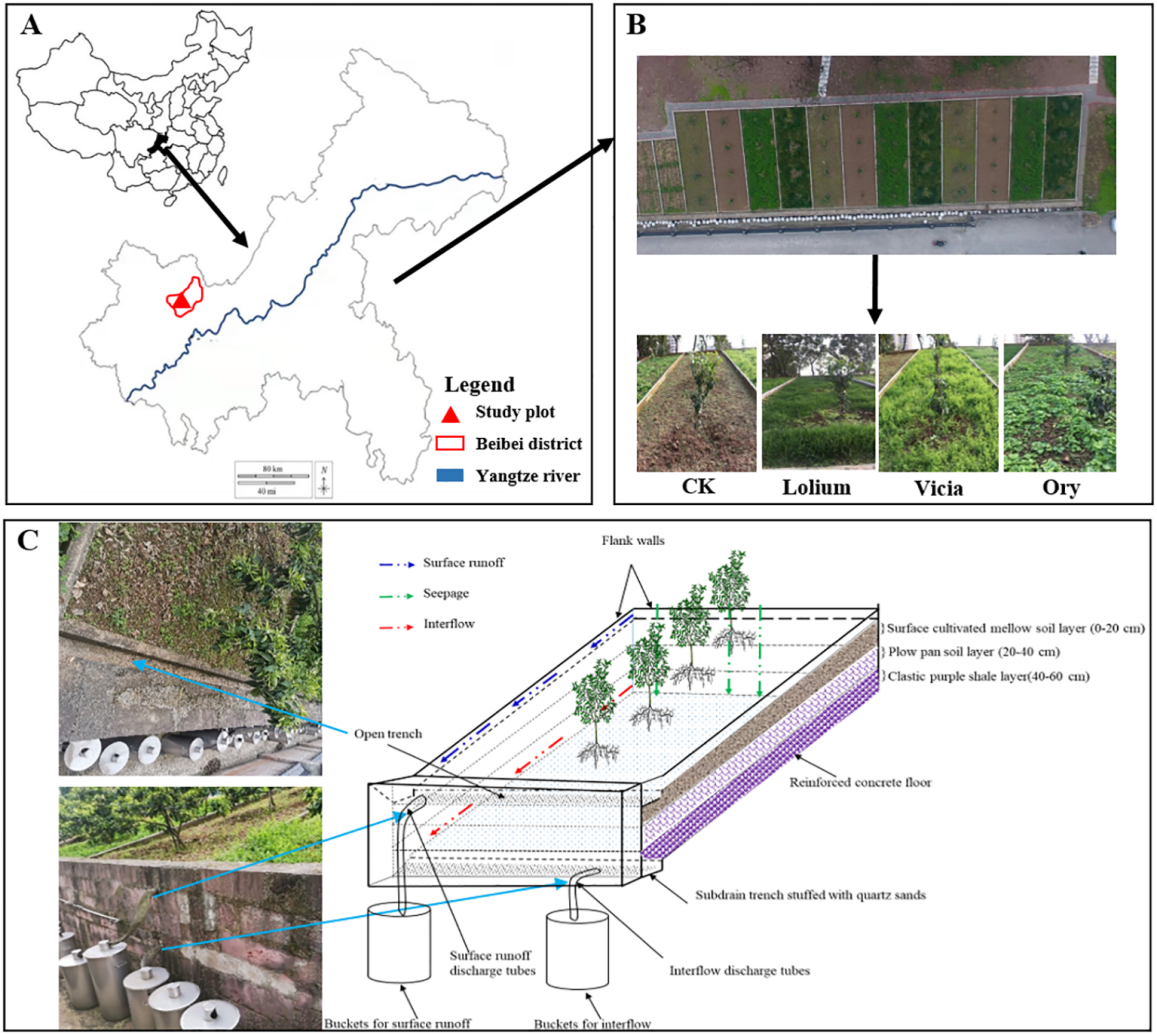
The location **(A)** and design **(B, C)** of the research plots.

The present field experiment was conducted in the runoff monitoring field of the National Monitoring Base for Purple Soil Fertility and Fertilizer Efficiency, which is located in Beibei, Chongqing, China (106°26′33″E, 29°48′36″N; altitude 266.3 m) (**Figure 1A**). This region has a subtropical monsoon climate with an average annual temperature of 18.3°C. The mean annual precipitation is 1115.3 mm, which is distributed unevenly through the year with April to August accounting for more than 80% of the total precipitation. The annual sunshine duration reaches 1276.7 h at an average annual frost-free period of 317 days (Zhang et al., 2021). The study region is dominated by purple soil formed in the purple sandy shale, classified as Entisol according to the USDA soil taxonomy. The physicochemical properties of the top soil layer were: pH 5.6, total N 1.2 g kg^-1^, total P 0.7 g kg^-1^, total potassium (K) 22.3 g kg^-1^, available N 98.4 mg kg^-1^, available P 55.8 mg kg^-1^, available K 90.7 mg kg^-1^, soil bulk density 1.22 g cm^-3^, and soil porosity 53.96%.

A total of 12 experimental plots were built on February 28, 2018 on a slope (**Figures 1B, C**). The size of each plot is 12 m (length) × 4 m (width) × 0.6 m (depth), with a slope of 15°, which allows the simultaneous monitoring of surface runoff and interflow (at 60 cm underground). The sidewalls and floors of the compartments were made of reinforced concrete to interrupt the connection of contiguous plots and to avoid hydrological interferences. The soil layers of each plot from top to bottom were 20 cm of surface-cultivated mellow soil, 20 cm of plow pan soil, and 20 cm of clastic purple shale parent materials, respectively, which originated from Ziyuntai, Beibei, Chongqing. The trench for the collection of surface runoff, sediment and interflow was placed at the bottom of each plot, and PVC pipes were used to connect each groove to the surface runoff and interflow collection barrel (**Figure 1C**).

In each runoff plot, four annual citrus seedlings of the *Citrus reticulata ‘Ai Yuan 38’* were transplanted in April 2018 into the central axis of each plot with a plant spacing of 3 m. The variety exhibits a moderate degree of polyembryony, and the seedlings are carefully selected for transplantation from uniformly sized seedlings grown under the same environmental conditions (**Figures 1B, C**). The fertilization level of each experimental plot was the same and amounted to 273 g N, 151 g P_2_O_5_, and 299 g K_2_O in 2018-2019, and 249 g N, 104 g P_2_O, and 178 g K_2_O in 2019-2020. The fertilizer was applied using the trenching method, where a shallow trench was dug around the drip line of the citrus trees, ensuring even nutrient distribution to the root zone. Soil nutrient analysis conducted at the end of the first year showed that residual nutrient levels were sufficient to support the continued growth of citrus trees (Chen et al., 2010). Additionally, as the trees matured, their nutrient requirements decreased compared to the seedling stage in the first year (Zhang et al., 2017). Furthermore, following local agricultural guidelines for citrus cultivation, fertilizer application rates were reduced in subsequent years to prevent excessive fertilization and minimize nutrient runoff. Therefore, the amount of fertilizer used in the second year was lower than in the first year. The selection of fertilization rates was based on local soil nutrient conditions and citrus tree growth stages, following standard agronomic practices. The prevention and control of citrus diseases and insect pests were carried out according to routine practices and the management of each plot was consistent (Lin et al., 2019).

### 2.2 Experimental design and collection of runoff samples

The field experiment was arranged in a randomized complete block design with four treatments, including: i) clean tillage as control (CK), which represents the habit of fruit farmers for keeping the ground bare by manual weeding or spraying herbicides; ii) coverage with *Lolium perenne* L. (Lolium), a gramineous grass; iii) coverage with *Vicia villosa* Roth (Vicia), a leguminous species; iv) coverage with *Orychophragmus violaceus* (Ory), a cruciferous species. Each treatment was set up in three replicates to ensure statistical validity. The *Lolium perenne* L., *Vicia villosa* Roth and *Orychophragmus violaceus* seeds were sown inside the plots in the middle of September 2018 and 2019 at rates of 22.5, 45.0, and 22.5 kg ha^-1^, respectively.

Runoff water samples were collected after natural rainfall events from September 2018 to September 2020. The water level in each container were measured using a ruler after each rainfall event to calculate the runoff flux. Subsequently, the runoff samples mixed with sediment were collected in a plastic bottle and dried in an oven to constant weight at 105°C to measure the sediment content (Ma et al., 2016). The product of runoff volume and sediment content was used to calculate the soil loss for each rainfall event. If soil was deposited in the confluence ditch, it was included in each treatment separately (Ma et al., 2016; Dai et al., 2018). Another runoff sample was collected in a 500 ml plastic bottle, immediately taken to the laboratory, and stored at 4°C for further chemical analyses. The main measurement parameters were various forms of nitrogen, i.e. total nitrogen (TN), soluble total nitrogen (DN), nitrate-nitrogen (NO_3_^–^N), ammonium nitrogen (NH_4_^+^-N), and various forms of phosphorus, i.e. total phosphorus (TP), soluble total phosphorus (DP), phosphate (PO_4_^+^-P). A 200 mL unfiltered aliquot was used to analyze the TN and TP content in each runoff sample, using alkaline potassium persulfate oxidation UV spectrophotometry and ammonium molybdate spectrophotometric, respectively (Ma et al., 2016; Xiong et al., 2022). The other runoff samples were filtered through a 0.45 μm membrane to analyze the concentrations of DN, NO_3_^–^N, NH_4_^+^-N, DP, and PO_4_^+^-P by a flow auto analyzer AA3 (Bran and Lubbe, Norderstedt, Germany) (Tian et al., 2017). The following formulas were used to determine particulate nitrogen (PN) and particulate phosphorus (PP): PN = TN - DN and PP= TP - DP (Li et al., 2017). Ground coverage by cover crops was quantified by photographing the cover crops at regular intervals, followed by digital image analysis to assess the extent of coverage (Bousselot et al., 2010). Meteorological data from 2018 to 2020 were acquired from the meteorological station of the National Monitoring Base for Purple Soil Fertility and Fertilizer Efficiency, approximately 50 m from the experimental site.

For each rainfall event, nutrient losses were calculated by multiplication of the runoff volume and nutrient content. Total annual nutrient losses were computed by the addition of these rainfall event values. The fluxes of loss of a certain form of N or P in a single rainfall runoff event were calculated as follows:


(1) Qi=Ci*qi/S*10

The annual flux of N or P loss (Q) under the rainfall events was calculated as follows (Zhang et al., 2016):


(2) $$\;Q=\sum_{i}^{n}Qi$$

Where Qi (g ha^-1^) represents the flux of loss of a certain form of N or P in surface runoff or interflow in the i-th rainfall, Ci (mg L^−1^) represents the contents of various forms of N and P in surface runoff and interflow water, qi (L) represents the surface runoff and interflow volume in a single event, S (m^2^) represents the area of the runoff plot, 10 represents the unit conversion factor (i = 1 to n, the number of runoff flow events during the year).

### 2.3 Determination of citrus growth parameters and collection of plant samples

Citrus tree height, stem diameter (near the ground surface) and crown width were recorded with a measuring tape and a vernier caliper on September 2018 (Prior to commencement of monitoring), 2019 (at the end of one year of monitoring) and 2020 (at the end of two years of monitoring). Changes in citrus tree parameters were obtained by subtraction. Mature leaves of spring shoots of citrus trees were collected from four directions (east, west, south, and north) in mid-May 2019 and 2020. The leaf dry weight was measured by heat treatment of leaves at 105°C for 15 minutes to inactivate the enzyme and then drying at 65°C for 72 h. The dried leaves were ground into powder by a grinder (JX-CL, Jingxin Ltd., Shanghai, China). Aliquots of 1.5 mg to 2.0 mg of dried powdered plant material were weighted into tin capsules combusted in an elemental analyzer (Flash EA, Thermo Fisher Scientific, Massachusetts, USA) connected to an isotope ratio mass spectrometer (253plus, Thermo Fisher Scientific, Massachusetts, USA) through a Conflo IV multi-purpose interface for C, N, and stable isotope natural abundance analysis, according the method described by Simon et al. (2011). Total P was determined by the molybdenum blue test with a spectrophotometer (UV-1800, AOE Instruments, Shanghai, China) as previously described (Netzer et al., 2017).

### 2.4 Collection of soil samples

In September 2019 and 2020, mixed soil samples (0-20 cm) from five spots in each plot were obtained using an auger after the removal of impurities on the surface. The collected soil samples were air-dried, ground, and then partly passed through 2.0 mm sieve and partly through a 0.25 mm sieve. The sieved and dried soil samples were analyzed for total N (TN), total phosphorus (TP) and organic matter (OM) contents as previously described (Cheng et al., 2019; Lu, 2000). Soil available N and available P were determined according to Olsen et al. (1982).

### 2.5 Statistical analysis

The raw data were first tested for normal distribution by Shapiro-Wilk tests. Where necessary, data were transformed using either log- or square-root transformation to satisfy the assumptions of normality and variance. One-way analysis of variance (ANOVA) followed by Duncan *post hoc* tests were employed to test the significances of difference between leaf nutrition, citrus growth and runoff, soil, and nutrient losses under the experimental treatments. All statistical tests were performed using with the SPSS 17.0 software (SPSS Inc., Chicago, USA). Differences were considered significant at *P*< 0.05. All the figures were generation of OriginPro 2016 (OriginLab Inc., Northampton, USA) and GraphPad Prism 6.0 (San Diego, CA, USA).

## 3 Results

### 3.1 Effectiveness of groundcover on nutrition and growth of citrus trees

**Figure 2:**
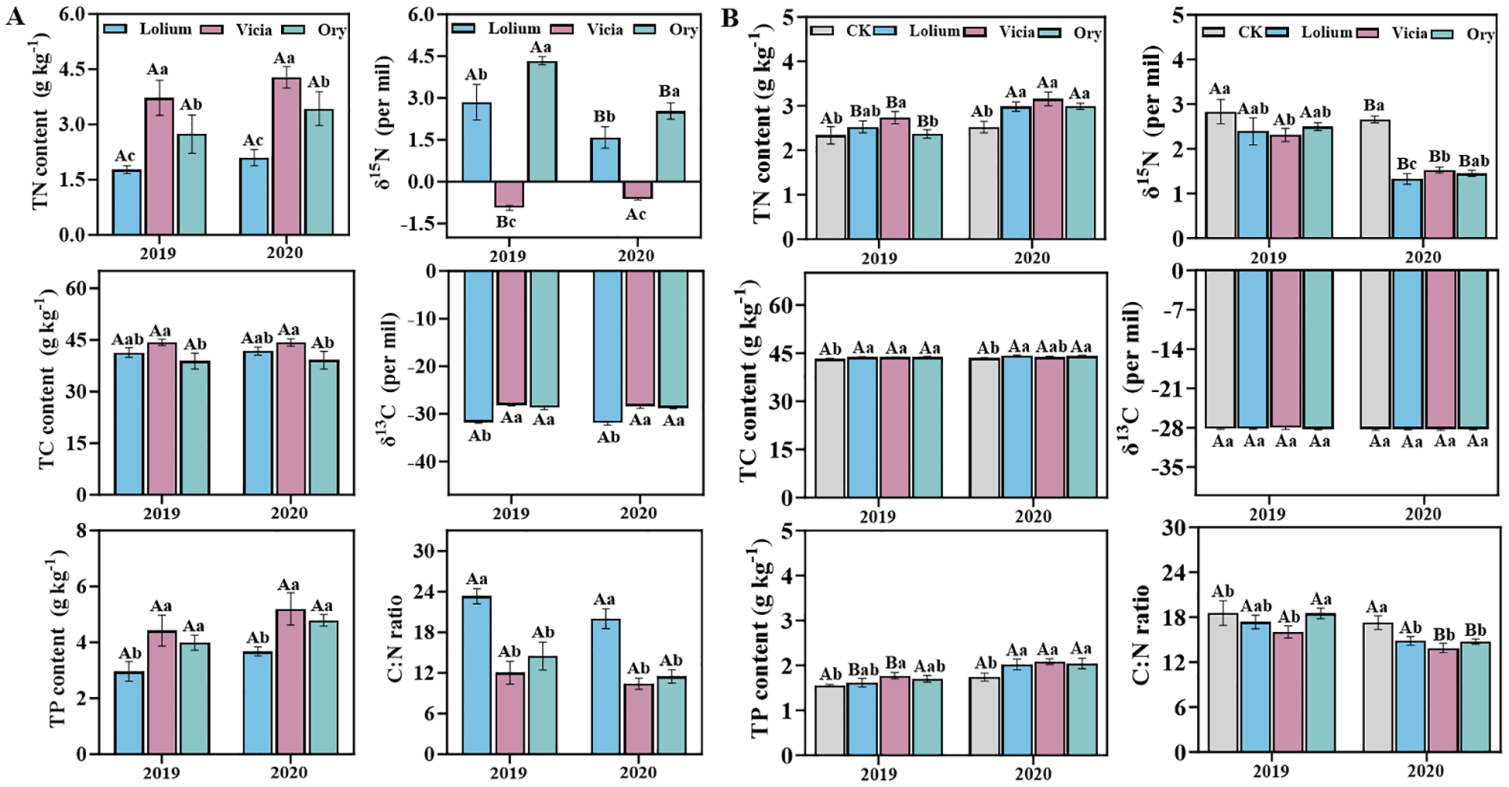
Total nitrogen content (TN), total carbon content (TC), total phosphorus content (TP) as well as C and N stable isotope signatures in **(A)** different groundcover species, and **(B)** the mature leaves of spring shoots of citrus trees as affected by different groundcover species. CK, clean tillage as control; Lolium, coverage with *Lolium perenne* L.; Vicia, coverage with *Vicia villosa* Roth Ory, coverage with *Orychophragmus violaceus*. Different lower-case letters (a-c) indicate significant differences between CK and groundcover management of the same experimental period. Different capital letters (A, B) indicate significant differences between the same treatment in different experimental periods.

Groundcover species mediated annual nursing effects on foliar nutrient contents of citrus trees (**Figures 2A, B**). In addition, different groundcovers significantly enhanced citrus tree growth and improve leaf nutrient (**Figure 2B**, **Table 1**). Specifically, significant positive effects on height, stem diameter and crown width of citrus trees were observed with the Vicia covering treatment within the first year of observation, but no significant effects on the number of mature leaves of spring shoots. After two consecutive years of groundcover management, Lolium and Vicia both significantly improved the growth of citrus trees, with increased height, stem diameter, and crown width by +19.92, +32.51, and +19.41%, (east-west direction by14.88% and north-south direction by 23.94%), and 37.20, 41.50, and 12.35% (east-west direction by17.74% and north-south direction by 6.95%), respectively compared to the CK treatment. The number of mature leaves of spring shoots was decreased, whereas total nitrogen (N), phosphorus (P) and carbon (C) contents of mature citrus leaves on spring shoots were increased at Lolium and Vicia ground cover. In addition, the total N and P contents of citrus spring leaves in 2020 were significantly higher than those in 2019, while the C/N and C/P ratios were reduced. In addition, groundcover significantly reduced the *δ* ^15^N natural abundance levels in mature leaves of citrus spring shoots compared to CK. In 2020 the foliar *δ* ^15^N natural abundance was more negative than in 2019 (**Figure 2B**). Groundcover had no significant effects on *δ* ^13^C natural abundance of citrus leaves (**Figure 2B**).

**Table 1:** Height, stem diameter and crown width increment of citrus tree as affected by different groundcover management.

Year	Treatments	Height increment (cm)	Stem diameter increment (mm)	Crown width increment (cm)	
				North-South	East-West
2019.09	CK	93.27 ± 19.71b	5.39 ± 1.16b	89.50 ± 21.31b	92.33 ± 13.21b
	Lolium	117.79 ± 20.69a	5.69 ± 1.89b	104.20 ± 19.70ab	100.09 ± 24.45a
	Vicia	119.52 ± 11.03a	7.59 ± 1.47a	117.23 ± 15.90a	103.00 ± 11.63a
	Ory	94.18 ± 118.58b	5.85 ± 1.71b	101.83 ± 11.83ab	107.11 ± 15.13a
2020.09	CK	114.90 ± 18.77c	14.06 ± 1.28b	147.18 ± 19.05b	115.58 ± 12.82b
	Lolium	137.79 ± 14.58b	18.64 ± 2.57a	169.08 ± 27.41a	143.25 ± 18.04a
	Vicia	157.64 ± 12.21a	19.90 ± 3.24a	157.42 ± 22.89a	136.08 ± 15.51a
	Ory	133.57 ± 113.60b	15.87 ± 1.79b	145.42 ± 20.56b	105.22 ± 13.85b

The lowercase letters (a - c) indicate significant differences of height, stem diameter, and crown width increment among different groundcover treatments at P ≤ 0.05. CK, clean tillage as control; Lolium, coverage with *Lolium perenne* L.; Vicia, coverage with *Vicia villosa* Roth; Ory, coverage with *Orychophragmus violaceus*.

### 3.2 Effectiveness of different groundcover species on soil nutrient contents

The present results showed that groundcover had no obvious effects on soil N and P nutrient contents after the first year (at the end of 2019), but at the end of the second year (2020), all groundcover treatments significantly increased total N (TN), total P (TP), available N (AN), available P (AP), and organic matter (OM) contents of the surface soil of the citrus orchard compared to CK by +26.35-51.10%, +22.56-32.36%, +21.57-54.72%, +9.26-28.68% and +10.77-18.57%, respectively (**Table 2**). In particularly, TN and AN contents of surface soil in the Vicia treatment were significantly higher than in the Lolium and Ory treatments (**Table 2**).

**Table 2:** Soil nutrient contents as affected by different groundcover management.

Year	Treatments	TN (g kg^-1^)	AN (mg kg^-1^)	TP (g kg^-1^)	AP (mg kg^-1^)	OM (g kg^-1^)
2019.09	CK	0.89 ± 0.01a	84.63 ± 6.97a	0.65 ± 0.04a	50.25 ± 4.91a	13.63 ± 0.61a
	Lolium	0.92 ± 0.03a	85.72 ± 3.10a	0.67 ± 0.06a	48.90 ± 5.26a	16.50 ± 1.11a
	Vicia	0.92 ± 0.03a	89.09 ± 2.65a	0.64 ± 0.03a	48.45 ± 4.63a	14.72 ± 3.16a
	Ory	0.89 ± 0.11a	85.45 ± 6.20a	0.69 ± 0.11a	43.65 ± 7.26a	14.87 ± 0.89a
2020.09	CK	0.74 ± 0.01c	69.73 ± 4.90c	0.57 ± 0.04b	45.41 ± 5.18c	13.86 ± 0.40b
	Lolium	0.93 ± 0.05b	87.19 ± 8.62b	0.68 ± 0.03a	49.61 ± 5.70bc	15.35 ± 0.84a
	Vicia	1.11 ± 0.03a	107.88 ± 7.94a	0.75 ± 0.03a	58.43 ± 2.19a	16.43 ± 1.02a
	Ory	0.94 ± 0.06b	84.77 ± 4.65b	0.69 ± 0.06a	52.83 ± 6.20ab	15.25 ± 0.61a

The lowercase letters (a - c) indicate significant differences of soil nutrient contents among different groundcover treatments at P ≤ 0.05. CK, clean tillage as control; Lolium, coverage with *Lolium perenne* L.; Vicia, coverage with *Vicia villosa* Roth; Ory, coverage with *Orychophragmus violaceus*.

### 3.3 Rainfall distribution and vegetation cover change

**Figure 3:**
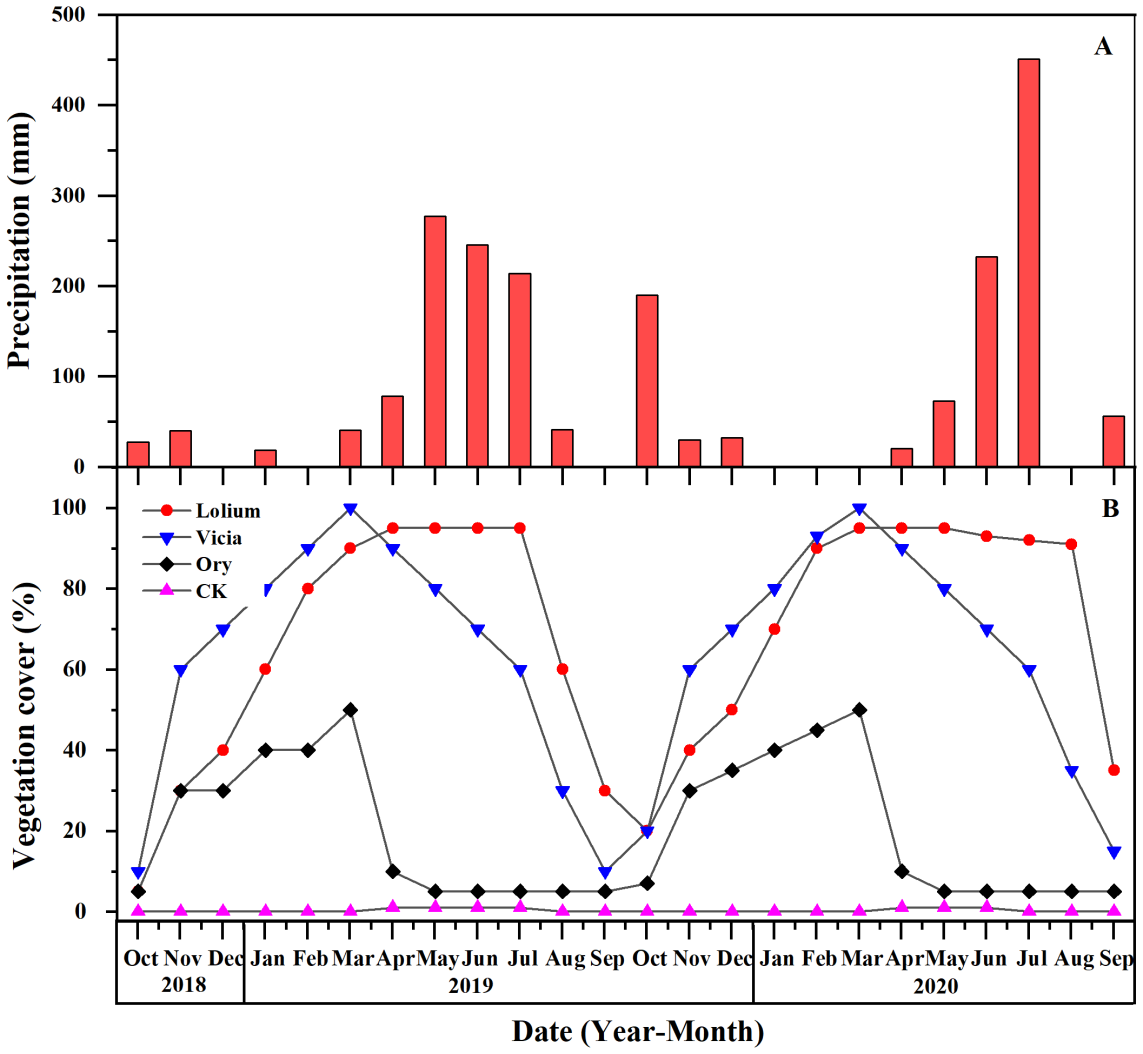
Monthly precipitation **(A)** and vegetation cover **(B)** of the soil during the monitoring period.

During the study period from September 2018 to September 2020, rainfall at the experimental site was mainly concentrated from May to July (**Figure 3A**). The total rainfall of the experimental plots between September 2018 and October 2019 was 980.66 mm and 75.05% of it was noted in May to July. The rainfall in May was highest, amounting to 276.90 mm and representing 28.25% of the total annual precipitation (**Figure 3A**). The total rainfall between September 2019 and September 2020 was 1,084.00 mm and 69.74% of it was noted in June to July. During this period, rainfall in July was highest, amounting to 451.00 mm and representing 41.61% of the total annual precipitation (**Figure 3A**). Cover crop type is a crucial factor controlling runoff and soil loss. During the study period, Lolium and Vicia had greater coverage on the ground, with Vicia reaching 60-100% coverage from November to March, while from April to July, Lolium had approached 95% coverage, greater than Vicia (**Figure 3B**). Ory came into full flowering in March with 50% ground cover, but from April to October with less than 10% ground cover (**Figure 3B**).

### 3.4 Surface runoff, interflow, and soil loss in the citrus orchard

**Figure 4:**
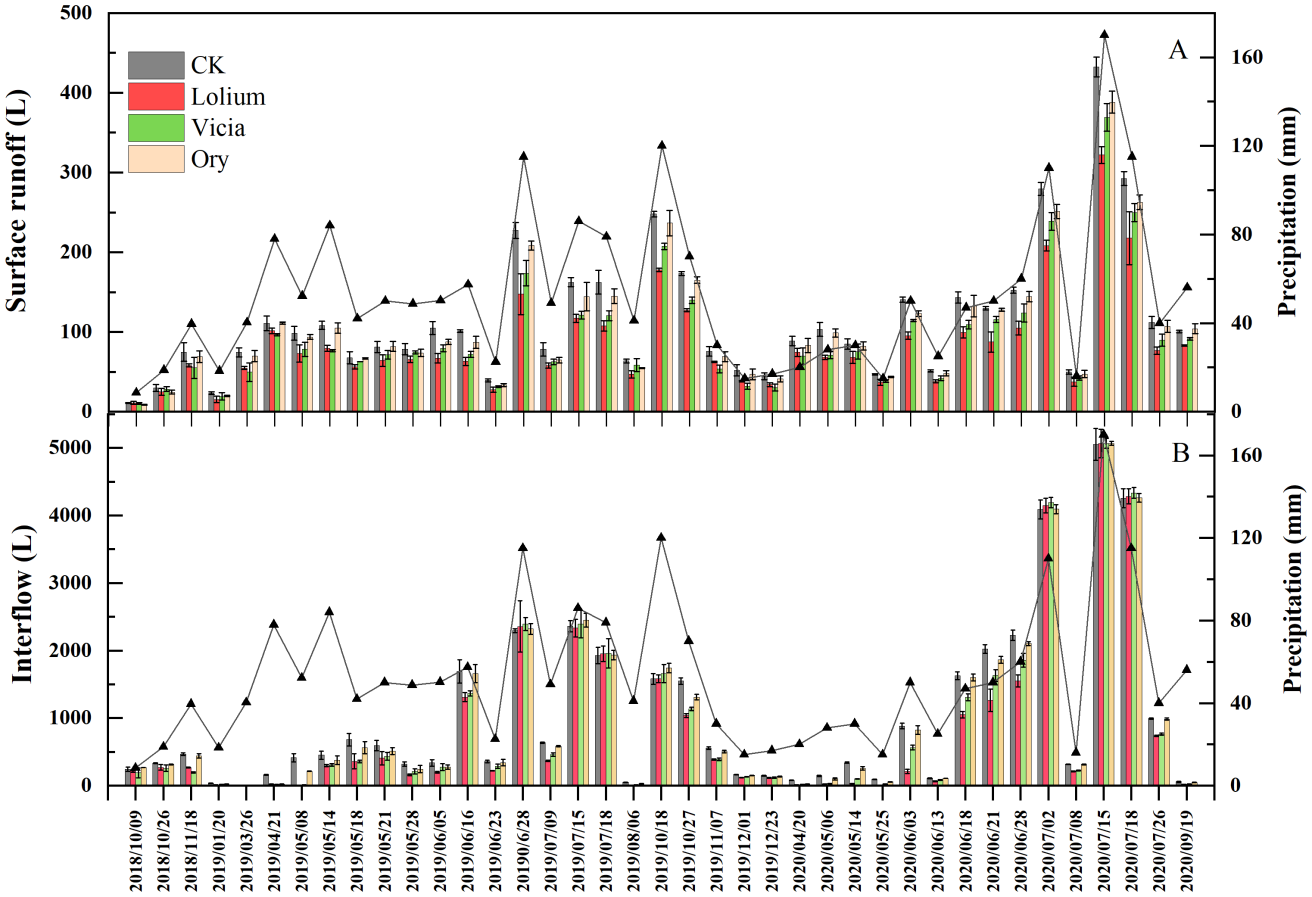
Dynamic changes of surface runoff **(A)** and interflow **(B)** with different groundcover management under rainfall events. Note: CK, clean tillage as control; Lolium, coverage with Lolium perenne L.; Vicia, coverage with *Vicia villosa* Roth; Ory, coverage with *Orychophragmus viola*.

Surface runoff, and interflow were collected to compare impacts of groundcover on these parameters. A total of 40 natural rainfall events generating surface runoff and 39 natural rainfall events producing interflow were measured during the period of the study (**Figure 4**). The amount of surface runoff and interflow of different treatments increased with precipitation. In the early stage of the experiment (September to October), there was no significant difference in surface runoff and interflow among the treatments. Since November, surface runoff from the groundcover treatments (especially Lolium and Vicia treatments) was smaller than that from the CK treatment. From April to July, the Lolium treatment had a better interception effect on the surface runoff, the interception effect was best in June and amounted to 25.41% - 37.69% less than in the CK treatment. The volumes of surface runoff collected from the Ory treatment were similar to the CK treatment. The interflow volume from the Lolium treatment was considerably smaller from April to May, but close to or greater than the CK treatment from June to July (**Figure 4**). Surface runoff and interflow peaked on July 15, 2020. Compared with the CK treatment, the surface runoff of Lolium, Vicia, and Ory treatments decreased by 25.51%, 14.61% and 10.18%, respectively, while the interflow increased by -0.29%, -0.34% and -0.31%, respectively (**Figure 4**).

The runoff and soil losses from orchards with groundcover were significantly smaller compared to the CK treatment (**Table 3**). The overall effect on the amount of surface runoff, interflow, and soil loss was Lolium > Vicia > Ory. The Lolium and Vicia plots showed a reduction of 27.17% and 18.94% in surface runoff, 18.50% and 13.60% in interflow, and 58.34% and 47.65% in soil loss (**Table 3**). In addition, the interflow was much higher than the surface runoff under the different treatments, and the average annual interflow amounted to 89.55% to 90.60% of the total annual runoff (**Table 3**).

**Table 3:** Annual surface runoff, interflow and soil loss caused by different groundcover management.

Year	Treatment	Precipitation mm	Runoff (mm)					Soil loss (kg ha^-1^)
			Surface runoff	Proportion of surface runoff (%)	Interflow	Proportion of interflow (%)	Total runoff	
2018.09- 2019.09	CK	980.7	35.4 ± 1.34a	11.3%	278.6 ± 0.74a	88.7%	314.0 ± 1.46a	317.3 ± 30.7a
	Lolium	980.7	25.8 ± 1.44c	10.3%	224.5 ± 1.95c	89.7%	250.4 ± 0.79d	157.0 ± 18.7
	Vicia	980.7	28.0 ± 0.78c	10.8%	232.3 ± 7.19c	89.2%	260.3 ± 7.06c	172.8 ± 36.3b
	Ory	980.7	32.3 ± 1.37b	11.0%	262.2 ± 8.92b	89.0%	294.5 ± 7.91b	288.6 ± 24.9a
2019.09- 2020.09	CK	1084	58.4 ± 0.43a	9.6%	547.9 ± 11.2a	90.4%	606.2 ± 10.8a	400.1 ± 14.7a
	Lolium	1084	42.4 ± 1.06d	8.5%	457.0 ± 3.96d	91.5%	499.5 ± 3.11d	135.4 ± 7.06d
	Vicia	1084	48.5 ± 1.11c	9.0%	493.0 ± 6.54c	91.0%	541.4 ± 6.10c	200.9 ± 16.3c
	Ory	1084	54.2 ± 1.32b	9.2%	532.6 ± 2.61b	90.8%	586.8 ± 2.06b	332.2 ± 23.3b

The lowercase letters (a - d) indicate significant differences of surface runoff, interflow, and soil loss among different treatments at *P* ≤ 0.05. CK, clean tillage as control; Lolium, coverage with *Lolium perenne* L.; Vicia, coverage with *Vicia villosa* Roth Ory, coverage with *Orychophragmus violaceus*.

### 3.5 Nitrogen losses by surface runoff and interflow

**Figure 5:**
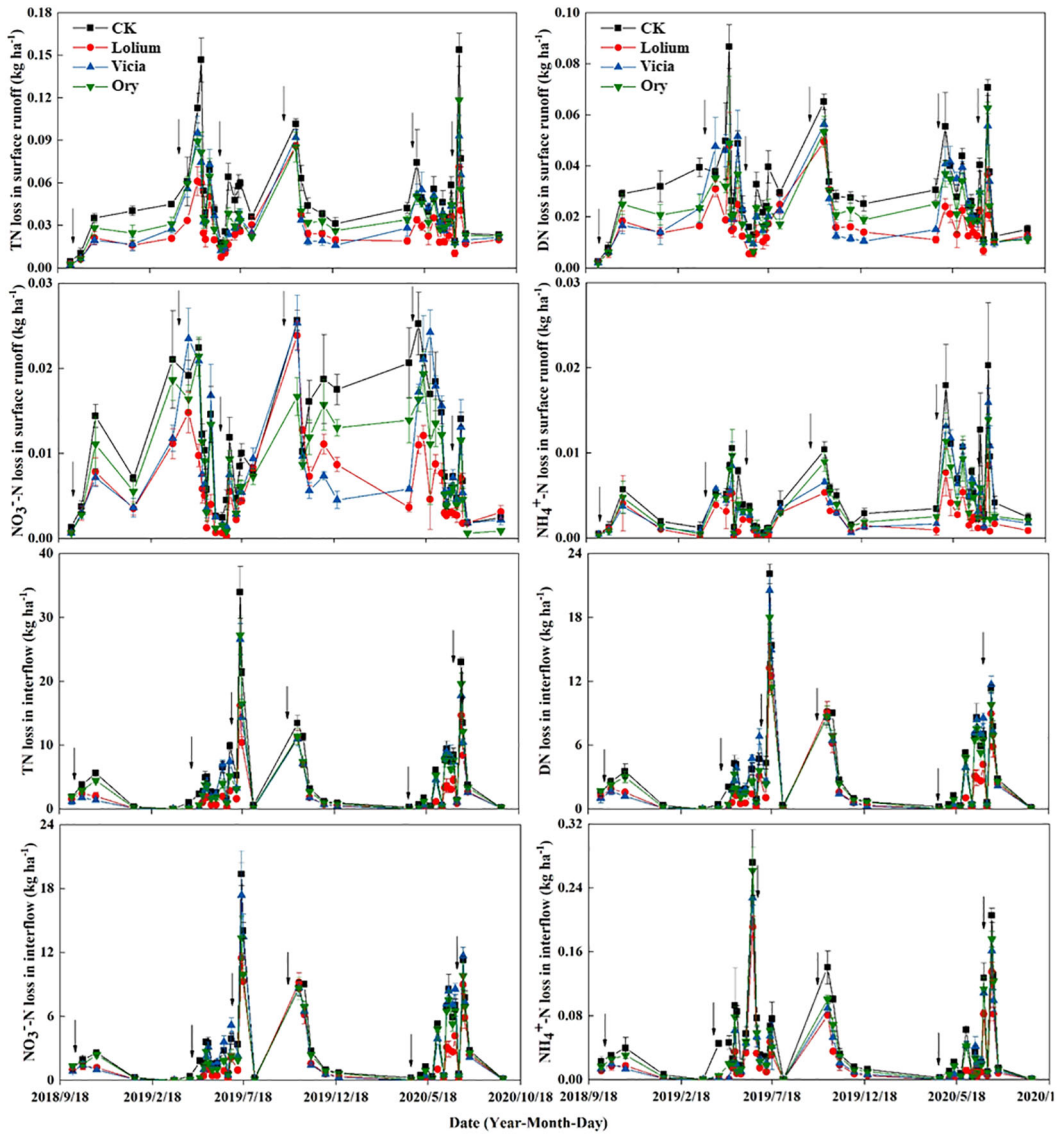
Dynamic changes of N loss in surface runoff and interflow with different groundcover management under rainfall events. CK, clean tillage as control; Lolium, coverage with *Lolium perenne* L.; Vicia, coverage with *Vicia villosa* Roth Ory, coverage with *Orychophragmus violaceus*. The vertical arrow indicates the fertilization time.

For the annual N loss dynamics, most of the results showed that the Lolium treatment reduced N losses more than the other groundcover treatments, followed by the Vicia treatment, whereas the effect of Ory treatment was minute (**Figure 5**). The loss of N in surface runoff was highest from April to June, while the loss of N in interflow was the highest from May to July (**Figure 5**).

The different groundcover treatments significantly reduced the annual loss of various forms of N in surface runoff and interflow compared to CK (**Table 4**). N losses via interflow were higher than those via surface runoff, averaging to 59.53-103.48 kg ha^-1^ per year, with significant differences in total N losses between groundcover treatments. In the Lolium treatment, surface runoff of TN, DN, NO_3_^–^N, NH_4_^+^-N and PN was reduced by 50.63%, 46.99%, 49.51%, 48.09%, and 56.18%, respectively, whereas the interflow was reduced by 50.51%, 46.40%, 50.50%, 50.93%, and 62.54%, respectively (**Table 4**). Furthermore, different groundcover treatments significantly reduced N fertilizer loss (**Table 4**). Compared with the CK treatment, Lolium, Vicia, and Ory treatment reduced the annual average N fertilizer loss by 42.74%, 14.10%, and 19.60%, respectively. There was no significant difference between the Vicia and Ory treatments. Overall, the Lolium treatment was most productive in decreasing the amount of N in surface runoff and interflow (**Table 4**).

**Table 4:** Forms of N loss (kg ha^-1^) in surface runoff and interflow and N fertilizer loss rate from different groundcover management.

Year	Treatments	Surface runoff					Interflow					N loss rate (%)
		TN	DN	NO_3_^–^N	NH_4_^+^-N	PN	TN	DN	NO_3_^–^N	NH_4_^+^-N	PN	
2018.09 - 2019.09	CK	0.97 ± 0.04a	0.58 ± 0.04a	0.18 ± 0.01a	0.07 ± 0.00a	0.38 ± 0.01a	101.91 ± 2.51a	75.94 ± 1.06a	64.02 ± 0.89a	1.00 ± 0.04a	25.97 ± 2.61a	45.22 ± 1.09a
	Lolium	0.48 ± 0.02c	0.31 ± 0.03c	0.09 ± 0.01c	0.03 ± 0.00c	0.17 ± 0.02d	50.44 ± 1.21d	40.71 ± 2.14d	31.69 ± 1.95d	0.49 ± 0.02c	9.73 ± 2.47d	22.38 ± 0.54d
	Vicia	0.65 ± 0.05b	0.43 ± 0.03b	0.14 ± 0.01b	0.05 ± 0.00b	0.21 ± 0.03c	82.35 ± 3.28b	66.11 ± 3.87b	55.06 ± 7.84b	0.64 ± 0.08b	16.24 ± 0.91c	36.48 ± 1.45b
	Ory	0.68 ± 0.04b	0.40 ± 0.03b	0.15 ± 0.00b	0.05 ± 0.00b	0.27 ± 0.02b	75.26 ± 4.27c	55.77 ± 2.22c	43.04 ± 3.20c	0.72 ± 0.04b	19.49 ± 2.10b	33.38 ± 1.87c
2019.09 - 2020.09	CK	1.04 ± 0.04a	0.65 ± 0.02a	0.26 ± 0.01a	0.15 ± 0.02a	0.38 ± 0.02a	107.06 ± 3.27a	80.84 ± 2.34a	57.50 ± 1.75a	0.90 ± 0.03a	26.22 ± 0.98a	52.07 ± 1.57a
	Lolium	0.62 ± 0.01c	0.38 ± 0.01c	0.14 ± 0.01c	0.06 ± 0.00d	0.23 ± 0.00d	69.71 ± 0.60c	49.24 ± 0.84c	37.43 ± 0.57c	0.58 ± 0.01c	20.47 ± 0.65b	33.88 ± 0.28c
	Vicia	0.80 ± 0.02b	0.52 ± 0.01b	0.21 ± 0.01b	0.12 ± 0.00b	0.28 ± 0.01c	97.57 ± 3.08b	75.03 ± 0.84b	50.93 ± 0.86b	0.78 ± 0.02b	22.54 ± 3.92ab	47.39 ± 1.49b
	Ory	0.83 ± 0.03b	0.53 ± 0.01b	0.19 ± 0.02b	0.10 ± 0.01c	0.31 ± 0.02b	94.22 ± 3.26b	71.17 ± 2.34b	49.86 ± 1.24b	0.76 ± 0.02b	23.05 ± 1.98ab	45.28 ± 1.85b

The lowercase letters (a - d) indicate significant difference of N loss in surface runoff and interflow and N fertilizer loss rate among different treatments at *P* ≤ 0.05. CK, clean tillage as control; Lolium, coverage with *Lolium perenne* L.; Vicia, coverage with *Vicia villosa* Roth Ory, coverage with *Orychophragmus violaceus*.

N was lost mainly in the form of DN (**Supplementary Table S1**). In the surface runoff, DN accounted for 59.69% - 66.99% of TN, and PN accounted for 33.01% - 40.31% of TN. In DN, NO_3_^–^N was the main component, accounting for 19.13% - 25.65% of TN (**Supplementary Table S1**). The DN in the interflow accounted for 66.02% - 80.74% of TN, and NO_3_^–^N accounted for 52.24% - 66.62% of TN. The Lolium and Vicia treatments significantly increased the loss of DN relative to PN. The Ory treatment did not significantly change the form of N lost in surface runoff and interflow (**Supplementary Table S1**).

### 3.6 Phosphorus losses in surface runoff and interflow

**Figure 6:**
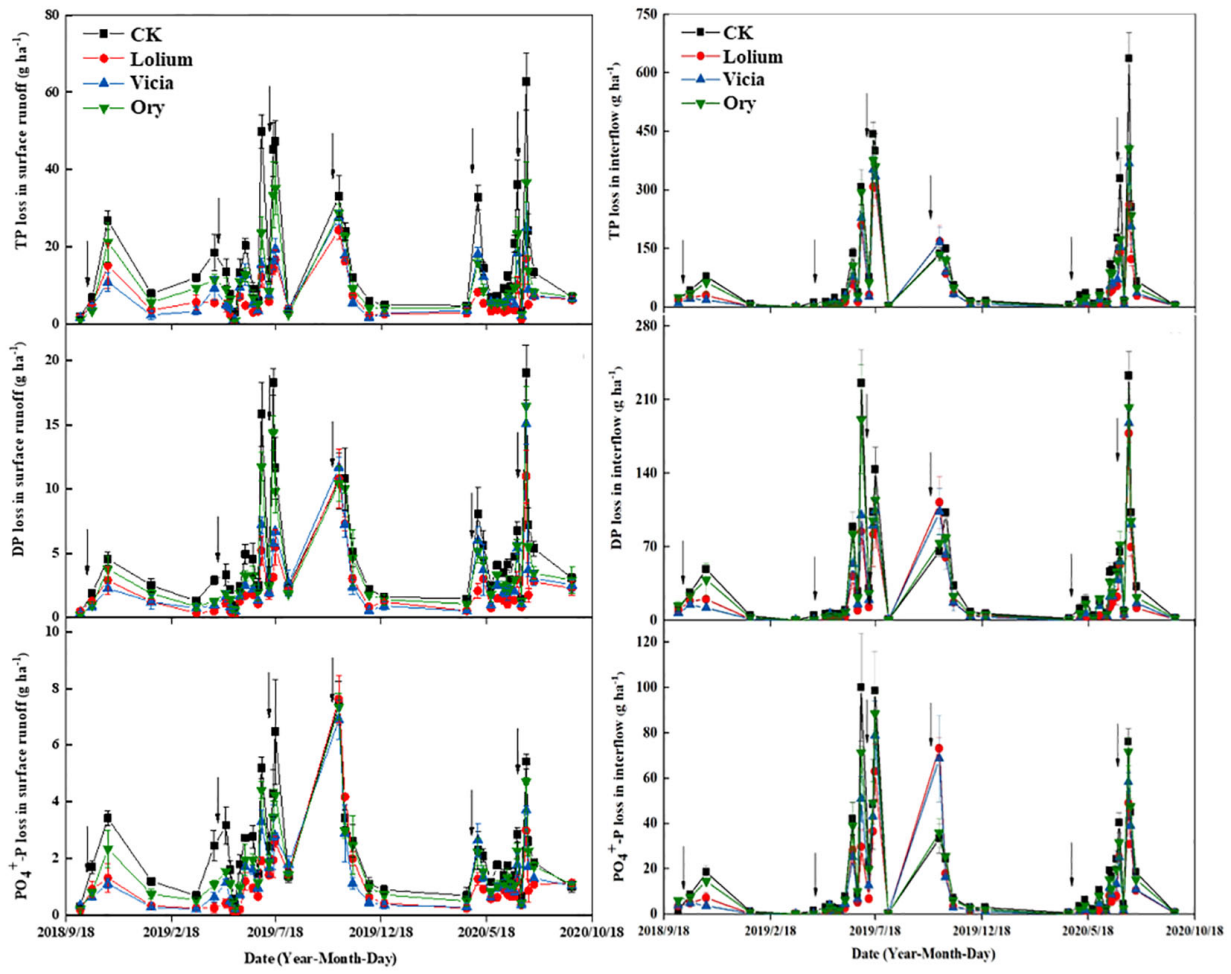
Dynamic changes of P loss in surface runoff and interflow with different groundcover management under rainfall events. CK, clean tillage as control; Lolium, coverage with *Lolium perenne* L.; Vicia, coverage with *Vicia villosa* Roth Ory, coverage with *Orychophragmus violaceus*. The vertical arrow indicates the fertilization time.

The interception effects of different treatments on the annual P loss in surface runoff and interflow differed between seasons (**Figure 6**). From October to March, most of the interception effects were in the order: Vicia > Lolium > Ory >CK. But from April to July, most of these effects were in the order: Lolium > Vicia >Ory >CK. At the initial stage of the experiment (October 2018), there was no significant difference in P loss among different treatments. In July, P loss was greater than that in other months (**Figure 6**). The losses of TP, DP, and PO_4_^+^-P in surface runoff and interflow were lowest under the Lolium treatment (**Figure 6**). The amount of TP, DP, and PO_4_^+^-P losses in surface runoff were 76.55, 29.87, and 12.34 g ha^-1^ for the Lolium treatment, respectively, with a reduction of 68.88%, 59.04%, and 53.60%, respectively, compared with CK (**Figure 6**). The amount of TP, DP, and PO_4_^+^-P losses in interflow was 1.20, 0.50, and 0.22 kg ha^-1^ for the Lolium treatment, respectively, with a reduction of 45.98%, 30.66%, and 37.00%, respectively, compared with CK treatment. Peak losses occurred from June to August (**Figure 6**).

Compared with the CK treatment, different groundcover treatments significantly reduced the loss of P in surface runoff and interflow (**Table 5**). The effects of the Lolium treatment on TP, DP, PO_4_^+^-P and PP in surface runoff and interflow were most significant (**Table 5**). The surface runoff of TP, DP, PO_4_^+^-P and PP was reduced by 61.52%, 55.03%, 48.16%, and 64.43%, respectively. The interflow of TP, DP, PO_4_^+^-P and PP was reduced by 46.09%, 41.54%, 39.35 and 48.16%, respectively (**Table 5**). Furthermore, different groundcover treatments significantly reduced the P fertilizer loss. Compared with the CK treatment, Lolium, Vicia, and Ory treatments reduced the annual average P fertilizer loss by 48.46%, 37.89%, and 23.42%, respectively (**Table 5**).

**Table 5:** Annual P loss (kg ha^-1^) in surface runoff and interflow and P fertilizer loss rate from groundcover management.

Year	Treatment	Surface runoff				Interflow				P loss rate (%)
		TP	DP	PO_4_^+^-P	PP	TP	DP	PO_4_^+^-P	PP	
2018.09 - 2019.09	CK	0.31 ± 0.01a	0.09 ± 0.00a	0.04 ± 0.00a	0.22 ± 0.01a	1.87 ± 0.07a	0.84 ± 0.04a	0.42 ± 0.06a	1.03 ± 0.04a	1.73 ± 0.06a
	Lolium	0.11 ± 0.01d	0.03 ± 0.00d	0.02 ± 0.00c	0.08 ± 0.01d	0.90 ± 0.08c	0.34 ± 0.06c	0.17 ± 0.01d	0.56 ± 0.03c	0.81 ± 0.07c
	Vicia	0.13 ± 0.01c	0.04 ± 0.00c	0.02 ± 0.00c	0.09 ± 0.01c	1.06 ± 0.10c	0.40 ± 0.02c	0.22 ± 0.01c	0.65 ± 0.08bc	0.95 ± 0.08c
	Ory	0.21 ± 0.01b	0.06 ± 0.00b	0.03 ± 0.00b	0.14 ± 0.01b	1.49 ± 0.21b	0.66 ± 0.06b	0.33 ± 0.04b	0.83 ± 0.17b	1.35 ± 0.16b
2019.09 - 2020.09	CK	0.34 ± 0.01a	0.11 ± 0.00a	0.04 ± 0.00a	0.23 ± 0.01a	2.39 ± 0.18a	0.94 ± 0.02a	0.37 ± 0.02a	1.45 ± 0.18a	3.14 ± 0.21a
	Lolium	0.13 ± 0.00d	0.06 ± 0.00d	0.03 ± 0.00b	0.08 ± 0.01d	0.92 ± 0.06c	0.53 ± 0.03d	0.23 ± 0.02d	0.39 ± 0.06d	1.22 ± 0.06d
	Vicia	0.19 ± 0.01c	0.08 ± 0.00c	0.03 ± 0.00b	0.12 ± 0.01c	1.21 ± 0.06d	0.62 ± 0.02c	0.25 ± 0.02c	0.59 ± 0.07c	1.61 ± 0.06c
	Ory	0.23 ± 0.01b	0.09 ± 0.00b	0.04 ± 0.00a	0.14 ± 0.01b	1.64 ± 0.02b	0.78 ± 0.03b	0.31 ± 0.02b	0.86 ± 0.02b	2.15 ± 0.01b

The lowercase letters (a - d) indicate significant differences of P loss in surface runoff and interflow and P fertilizer loss rate among different treatments at *P* ≤ 0.05. CK, clean tillage as control; Lolium, coverage with *Lolium perenne* L.; Vicia, coverage with *Vicia villosa* Roth Ory, coverage with *Orychophragmus violaceus.*

The loss of P was mainly in the form of PP (**Supplementary Table S2**). In surface runoff, PP accounted for 57.63% - 71.77% of TP, and DP accounted for 28.23% - 42.37% of TP, while PO_4_^+^-P only accounted for 12.66% - 21.10% of P loss. The PP in interflow accounted for 43.06% - 63.07% of P loss by TP (**Supplementary Table S2**). The proportion of PP loss in interflow was less than that in surface runoff (**Supplementary Table S2**). Groundcover significantly increased the contribution of DP and decreased the contribution of PP to P loss in the second year of the study (September 2019 - September 2020) (**Supplementary Table S2**).

## 4 Discussion

### 4.1 Groundcover improves nutrition and growth of citrus trees

The present study shows that groundcover can greatly improve soil nutrient contents and growth of citrus trees in orchards and can enhance the foliar nutrient content of citrus trees (**Figure 2**, **Tables 1**, **2**). This result is consistent with our hypothesis (i) and previous reports showing that groundcover can improve soil properties (Zhou et al., 2020; Tu et al., 2021). However, information on the consequences of groundcover for growth and nutrition of citrus trees has so far not been provided. After two consecutive years of groundcover, the legume species (*Vicia villosa* Roth) showed greater effectiveness than the non-legume species (*Lolium perenne* and *Orychophragmus violaceus*) in improving soil properties and foliar nutrient contents of citrus trees. This may be due to biological N_2_ fixation (BNF) by the legumes, which in turn promoted the growth of citrus trees. Also in previous studies on other species it has been reported that BNF by co-cultivated legumes can promote nutrient availability and growth of non-N_2_-fixing species, especially when soil N availability is limited. Co-cultivation N_2_-fixing *Robinia pseudoacacia* and non-N_2_-fixing *Juglans regia* in a mixed stand significantly improved soil nutrient availability and in turn increased N and P content as well as root biomass in non-N_2_-fixing *Juglans regia* (Hu et al., 2017; Du et al., 2019). Similarly, Sayyad et al. (2006) also reported a significant increase in foliar N concentration for a non-N_2_-fixing *Populus deltoides* in mixed cultivation with N_2_-fixing *Alnus subcordata*. *δ* ^15^N abundance is closely correlated to BNF, with lower *δ* ^15^N abundances in N_2_-fixing than in non-N_2_-fixing plants (Hu et al., 2017). In this study the *δ* ^15^N abundance was more negative in the legume crop (*Vicia villosa* Roth) compared to the non-legume cover crops investigated (*Lolium perenne* L. and *Orychophragmus violaceus*), which coincided with enhanced total N content in both, the legume crop tissues and citrus tree leaves (**Figure 2**). These results indicate that N_2_ fixed by *Vicia villosa* Roth contributed to the N nutrition the citrus trees. This conclusion is supported by the positive correlation of soil and foliar *δ* ^15^N, indicating that increased soil N and enhanced N nutrition of the citrus trees. However, Nikiema et al. (2012) showed that in a newly established *Abies fraseri* (Fraser fir) plantation cover crops competed with the major tree species for nutrients and water. As a consequence, the N and P contents of Fir leaves were reduced after 2 years of cover management. This difference to the present field study can be attributed to differences in management, climate or water availability (Cherr et al., 2006). Apparently, efficient nutrient uptake by the fir trees was prevented by dry weather conditions in the study by Nikiema et al. (2012).

### 4.2 Effects of groundcover on surface runoff, interflow, and soil loss

Our results indicate that groundcover on purple soil land slopes can significantly reduce surface runoff and soil loss (**Table 3**). This result is consistent with our hypothesis (ii) and previous results of Liu et al. (2012) obtained in a citrus orchard on yellow cinnamon soil in a reservoir area of central China. In agreement with these findings, also previous reports on other soil types and/or production systems indicated that the application of groundcover management in the inter rows is one of the most effective soil management practice to prevent soil erosion in sloping orchards (Morvan et al., 2014; Ferreira et al., 2018; Bagagiolo et al., 2018), thereby improving also other ecosystem services, such as rainwater interception and absorption of runoff energy by the soil surface (Montanaro et al., 2017; Feng et al., 2018; Winter et al., 2018). In addition, Almagro et al. (2016) reported that groundcover reduced runoff and soil loss by 62.3% and 56.3%, respectively, in an avocado orchard on Calcisol soil on a farmland slope of southeastern Spain while research of citrus orchards in a red soil region of southern China, revealed 89.4% and 99.7% reduction in runoff and soil loss (Mo et al., 2019). Such differences between different field studies may be associated with local climate conditions, soil properties, landscape structure (plain and sloping farmland with different inclination and slopes), and growth of different groundcover species (Deng et al., 2019a; Martinez-Mena et al., 2020). The selection of crop species in the present experiment was based on the previous study by Yang (2020) showing the potential of *Lolium perenne* L. (Lolium), *Vicia villosa* Roth (Vicia) and *Orychophragmus violaceus* (Ory) in citrus orchards for runoff and nutrient interception. As expected by our hypothesis (iv), the variations between groundcover systems in the present study were closely related to growth, development, and the degree of coverage of the crop species applied. Compared to Lolium and Vicia the Ory treatment had a poorer interception effect on surface runoff and soil loss (**Figure 4**, **Table 3**). This was probably due to the long surface coverage period, higher amounts of surface coverage and growth conditions of Lolium that reduced runoff and soil erosion. Additionally, Vicia began to decompose in April, and the surface coverage decreased to about 60% in July, whilst Ory had higher requirements for soil fertility and had the lowest surface coverage of about 40% to 50% in March, and less than 10% after April among three crop species studied (**Figure 3B**). Other investigations also highlight that more vegetation coverage can intercept higher amounts of runoff and enhance the infiltration of rainwater into the ground, effectively reduce the splashing of rain on the soil surface, and that parts of the sediment particles carried by the runoff could be intercepted by grass stems, thus restricting soil erosion (Deng et al., 2019b; Zhu et al., 2021; Liu et al., 2021). The results of Wang et al. (2019a) also demonstrated that the energy of rainfall was influenced by differences in vegetation coverage, which was the main factor affecting soil loss. In this context, compared to Vicia, Lolium was a more effective buffer at the soil surface due to its higher density of stems and larger biomass at enhanced surface coverage (**Figure 3**), thereby decreasing the speed and energy of surface runoff and further reducing soil erosion.

The present results indicated that interflow was one of the main pathways of runoff from purple soil and that the annual interflow accounts for 89.6% - 90.6% of total runoff (**Figure 4**, **Table 3**), in line with our hypothesis (iii) and Hua et al. (2014). It can be predicted that due to the dual geological structure of purple soil with surface soil and underlying rocks, affluent soil pores, intense penetration, and inefficient water holding capability may cause severe interflow losses under intensive rainfall and high temperatures (Qian et al., 2020; Khan et al., 2016), especially on farmland slopes (Xie et al., 2018). Our results show that on the early plant developmental stage, effects of different groundcover systems on surface runoff and interflow were similar to the control (**Figure 4**). However, with increasing time of groundcover cultivation, amounts of surface runoff and interflow changed oppositely (**Figure 4**). This is assumed to be due to the small amount and low intensity of rainfall in the early stage of plant development (September to April of the next year). Under this condition, the aboveground part of the vegetation could effectively intercept rainfall and reduce surface runoff (Liu et al., 2021). Previous studies have reported the root systems had a good winding and consolidation effect on the soil, by increasing soil porosity, reducing soil bulk density and, hence, enhancing the stability of soil aggregates and soil cohesion (Duan et al., 2020; Liu et al., 2021). This way, the ability of water retention and soil consolidation was enhanced, ultimately mediating high soil and water retention and reducing both, surface runoff and interflow. On the other hand, June to July is the annual rainy season in the local area with high amounts and intensity of rainfall. The highest individual rainfall observed was 170 mm, which led to soil saturation and increased interflow. Although the vegetation coverage significantly reduced the surface runoff, the interflow was increased due to the large root systems and cracks were easily generated in the soil to cause more interflow. Previous studies reported that rainwater produces surface runoff when its intensity exceeds the intensity of soil infiltration that is changed by the vegetation coverage (Huang et al., 2013). The stems and leaves of vegetation can intercept rainfall, while the roots form irregular soil voids, thereby increasing soil permeability (Zhang et al., 2020; Liu et al., 2021).

### 4.3 Groundcover reduces N and P losses in runoff and interflow

Nitrogen (N) and phosphorus (P) can move from soil to water by dissolving in surface runoff or interflow, thereby inducing non-point source pollution to the surrounding area (Cameron et al., 2013; Zhang et al., 2020). Groundcover could control N and P losses not only by minimizing the destruction of soil structure caused by raindrops and decreasing the surface runoff velocity (Yagioka et al., 2015; Keesstra et al., 2019), but also by efficient nutrient uptake by the root system of the cover crops (Solangi et al., 2019; Tran et al., 2021). Previous studies in grassland and farmland slopes established in other soil types reported that groundcover improved soil porosity, accelerates the formation of macro-aggregates (Villarreal et al., 2021), and decreased the risk of non-point source pollution (Griffith et al., 2020). The present results indicated that different groundcover in citrus orchards on farmland slopes on purple soil could significantly reduce N and P losses in the surface runoff and interflow, controlled fertilizer losses, and further improved soil fertility to promote the growth of young citrus trees as hypothesized (hypothesis i, ii) (**Tables 1**, **2**, **4**, **5**). Groundcover improves nutrient use efficiency by root uptake and enhances soil retention (Liu et al., 2021). In addition, cover crops could also add organic compounds to the soil by root exudation that supports soil decomposition and mineralization processes (Zhang et al., 2020; Maurer et al., 2021). In agreement with our hypothesis (iv), the capacity of cover plants to decrease nutrient losses is highly dependent on the vegetation species selected. Regarding runoff, N and P losses, our results showed that the Lolium has the most positive effect, followed by Vicia (**Tables 4**, **5**). This outcome may be due to the fact that leguminous (Vicia) could fix atmospheric N_2_ and thus induce a net gain of soil N (Xie et al., 2016). This conclusion is supported by the data on *δ* ^15^N in plants and total N in soil in the present study. Apparently, in the present study, the legume plant (Vicia) had a more negative *δ* ^15^N signal compared to the graminaceous plant (Lolium) and the cruciferous plant (Ory). In addition, the low C/N ratio of degrading plant material may result in rapid N decomposition, whereas the gramineous Lolium with a relative high C/N rate of degrading plant material may result in low N mineralization and slow N release to the soil (Zhou et al., 2020, Zhou et al., 2019). This partially explains a greater reduction of N and P losses with the gramineous Lolium from May to June. Although Vicia increased the loss of N and P during the decomposition period (from April to June) compared to the graminaceous plant, the total annual loss of N and P in the runoff was significantly lower than that of the clean tillage control treatment, because dead branches and residues of Vicia still seemed to effectively control runoff. Overall, Lolium is a beneficial cropping system of the present study to reduce soil erosion in citrus orchards on sloping land of purple soil. However, in terms of long-term orchard sustainable nutrient management systems, the legume Vicia has wider potential.

Our results indicated that different vegetation cover crops did not show preferable forms of N and P losses through runoff and interflow (**Supplementary Tables S1**, **S2**). Overall, total soluble nitrogen (DN) was the dominant form of N lost in surface runoff and interflow for all investigated cover crop species. Because nitrate-nitrogen (NO_3_^–^N) is the dominant form of DN, the loss of NO_3_^–^N was much higher than the loss of ammonium nitrogen (NH_4_^+^-N) (**Supplementary Table S1**). This finding is consistent with previous research on the effect of long-term fertilizer management on nutrient loss from purple soil slopes in Southwest China by Du et al. (2021) and on the mechanism of N and P losses from plain farmland (soil was mostly formed by alluvial flood, including tidal soil, meadow cinnamon soil, and calcareous cinnamon soil) in the North China Plain by Liu et al. (2014). Apparently, NH_4_^+^-N is readily absorbed by negatively charged particulates in the soil, whereas NO_3_^–^N is mostly present in soil solution and easily lost with runoff at heavy precipitation (Chen et al., 2012; Baptista et al., 2015). In addition, in the rhizosphere of citrus trees, microbial activities such as nitrification will rapidly transform reduced forms of N in the fertilizer into NO_3_^–^N that is easily absorbed and leached to lower layers of the soil profile (Chen et al., 2019).

In the present study, soluble P losses amounted to 28% - 57% of the total phosphorus (TP) losses (**Supplementary Table S2**), indicating that particulate P was the dominant form of P loss from the soil. This is due to the fact that phosphate has a high affinity to soil minerals and is readily enclosed and immobilized by soil particles (Wang et al., 2019b; Du et al., 2021). In addition, the amount of N and P losses in interflow accounts for a considerable share of surface runoff in steep slopes [Han et al., 2010; Tan and Zhang, 2011, hypothesis (ii)]. Therefore, for purple soil developed on farmland slopes, controlling runoff is an important means to reduce N and P losses, with the generation of interflow being particularly important to control nutrition losses.

## 5 Conclusion

The present study quantified the water, soil and nutrients retention, in particularly through both surface runoff and interflow pathways of soil by groundcover management on sloping farmland systems in purple soil regions. The presence of groundcover significantly reduced surface runoff, interflow, soil erosion as well as N and P losses compared to clean tillage. In addition, the enhanced growth and improved leaf N and P nutrient contents of citrus tree were observed with the groundcover systems. However, the effectiveness of such effects highly depended on the species of groundcover. The Lolium treatment was more effective in controlling soil and water losses and reducing N, P losses from both runoff and interflow than other treatments. Compared with clean tillage, ground coverage with Lolium can reduce soil, N and P losses by 216.46 kg ha^-1^, 44.87 kg ha^-1^ and 1.11 kg ha^-1^, respectively. From these results, it is estimated that the annual reduction potentials of soil, N and P losses that could be obtained by groundcover amounts to 16.3 million tons soil yr^-1^, 3.44 million tons N yr^-1^and 8.5 million tons P yr^-1^, respectively on average based on the areas of purple soil slope farmland in Southwestern China. For such reductions, Lolium constitutes the more beneficial coverage system to reduce soil, water, and nutrient losses from surface runoff and interflow in the citrus orchards. However, cultivation of the legume Vicia significantly enhanced soil nutrition status in citrus orchards and promoted leaf nutrients levels, which further promoted the growth of young citrus trees. In terms of a sustainable nutrient management system for long-term orchards, the leguminous Vicia also has the great potential to bring economic and ecological values for rehabilitation of the vulnerable and eroded sloping farmland systems in purple soil regions. Thus, the selection of appropriate groundcover to match the target of improved soil properties and/or facilitative effects on nutrient status of citrus trees is essential for the sustainable development of citrus orchard, particularly on sloping farmland systems. Further effects of groundcover management on the yield and fruit quality of citrus should be considered in future studies.

## Data Availability

The original contributions presented in the study are included in the article/**Supplementary Material**. Further inquiries can be directed to the corresponding author.
