# Supplementary material for: Groundcover improves nutrition and growth of citrus trees and reduces water runoff, soil erosion and nutrient loss on sloping farmland
Source: Front Plant Sci. 2024 Nov 6;15:1489693. doi: 10.3389/fpls.2024.1489693 (PMC11576175; doi:10.3389/fpls.2024.1489693)
Supplement: Supplementary file 1 [file Table1.docx]

**Supplementary Material:**

**Supplementary Table S1.** Percentage of different forms of N losses in surface runoff and interflow as affected by different groundcover management.

| Year | Treatments | Surface runoff（%） | | | |  | interflow（%） | | | |
| --- | --- | --- | --- | --- | --- | --- | --- | --- | --- | --- |
|  |  | DN/TN | NO_3_^-^-N/TN | NH_4_^+^-N/TN | PN/TN |  | DN/TN | NO_3_^-^-N/TN | NH_4_^+^-N/TN | PN/TN |
| 2018.09 - | CK | 60.43±0.99ab | 19.13±0.57a | 6.91±0.33a | 39.57±0.99ab |  | 74.59±2.02b | 62.83±0.69a | 0.98±0.06a | 25.46±2.02a |
| 2019.09 | Lolium | 64.88±3.96ab | 19.59±2.13a | 7.29±1.25a | 35.12±3.96ab |  | 80.74±4.66a | 62.91±5.32a | 0.97±0.04a | 19.26±4.66b |
|  | Vicia | 66.99±1.48a | 21.91±0.92a | 7.50±0.87a | 33.01±1.48b |  | 80.24±1.67a | 66.62±6.98a | 0.78±0.08b | 19.76±1.67b |
|  | Ory | 59.69±2.48b | 22.12±1.90a | 7.93±0.54a | 40.31±2.48a |  | 74.16±1.33b | 57.16±1.56a | 0.96±0.05a | 25.84±1.33a |
| 2019.09 - | CK | 63.14±0.68b | 25.06±0.19a | 14.25±1.33a | 36.86±0.68a |  | 80.41±0.63a | 53.74±1.98a | 0.84±0.04a | 19.59±0.79c |
| 2020.09 | Lolium | 61.95±0.83b | 22.68±1.00b | 9.44±0.18c | 38.05±0.89a |  | 66.02±0.83c | 53.70±0.70a | 0.83±0.02a | 33.98±1.05a |
|  | Vicia | 65.80±0.76a | 25.65±0.16a | 14.86±0.10a | 34.20±0.76b |  | 72.83±3.25b | 52.24±2.29a | 0.80±0.01a | 27.17±3.31b |
|  | Ory | 63.18±0.83b | 23.38±1.46b | 12.33±0.50b | 36.82±0.83a |  | 77.10±1.43a | 52.95±1.76a | 0.81±0.05a | 22.90±1.42c |

Note: The lowercase letters (a - d) indicate significant difference of N loss in surface runoff and interflow and N fertilizer loss rate among different treatments at P ≤ 0.05. CK, clean tillage as control; Lolium, coverage with *Lolium perenne* L.; Vicia, coverage with *Vicia villosa* Roth var.; Ory, coverage with *Orychophragmus violaceus*.

**Supplementary Table S2.** Percentage of different forms of P losses in surface runoff and interflow as affected by different groundcover management.

| Year | Treatment | Surface runoff（%） | | |  | interflow（%） | | |
| --- | --- | --- | --- | --- | --- | --- | --- | --- |
|  |  | DP/TP | PO_4_^+^-P/TP | PP/TP |  | DP/TP | PO_4_^+^-P/TP | PP/TP |
| 2018.09- | CK | 28.23±1.59b | 14.31±0.33b | 71.77±1.59a |  | 45.15±1.96a | 22.52±2.51ab | 54.85±1.96b |
| 2019.09 | Lolium | 28.44±0.76b | 14.37±0.80b | 71.56±0.76a |  | 36.93±3.75b | 18.73±2.02b | 63.07±3.75a |
|  | Vicia | 31.40±1.52a | 16.90±0.88a | 68.60±1.52b |  | 38.20±1.70b | 21.09±.89ab | 61.80±1.70a |
|  | Ory | 31.04±1.49a | 14.70±0.75b | 68.96±1.49b |  | 45.51±4.28a | 23.00±2.15a | 54.49±4.28b |
| 2019.09 - | CK | 32.11±0.99b | 12.66±0.18c | 67.89±0.99a |  | 39.57±3.04c | 15.48±0.81c | 60.43±3.04a |
| 2020.09 | Lolium | 42.37±3.33a | 21.10±0.72a | 57.63±3.33b |  | 56.94±4.92a | 23.68±1.30a | 43.06±4.92c |
|  | Vicia | 39.67±2.85a | 16.37±0.66b | 60.33±2.85b |  | 50.54±4.05ab | 20.58±1.56b | 49.46±4.05bc |
|  | Ory | 39.13±1.06a | 16.40±0.48b | 60.87±1.06b |  | 47.23±1.20bc | 19.17±1.16b | 52.77±1.20ab |

Note: The lowercase letters (a - d) indicate significant differences of P loss in surface runoff and interflow and P fertilizer loss rate among different treatments at *P* ≤ 0.05. CK, clean tillage as control; Lolium, coverage with *Lolium perenne* L.; Vicia, coverage with *Vicia villosa* Roth var.; Ory, coverage with *Orychophragmus violaceus.*
